# Predicting knee osteoarthritis risk in injured populations

**DOI:** 10.1016/j.clinbiomech.2017.06.001

**Published:** 2017-08

**Authors:** Michael J. Long, Enrica Papi, Lynsey D. Duffell, Alison H. McGregor

**Affiliations:** a Department of Surgery and Cancer, Imperial College London, Room 7L16, Floor 7, Laboratory Block, Charing Cross Hospital, London W6 8RF, UK; b Department of Medical Physics and Biomedical Engineering, University College London, Gower Street, London WC1E 6BT, UK

**Keywords:** Osteoarthritis, Knee, Injury, Gait, K-Nearest Neighbour

## Abstract

**Background:**

Individuals who suffered a lower limb injury have an increased risk of developing knee osteoarthritis. Early diagnosis of osteoarthritis and the ability to track its progression is challenging. This study aimed to explore links between self-reported knee osteoarthritis outcome scores and biomechanical gait parameters, whether self-reported outcome scores could predict gait abnormalities characteristic of knee osteoarthritis in injured populations and, whether scores and biomechanical outcomes were related to osteoarthritis severity via Spearman's correlation coefficient.

**Methods:**

A cross-sectional study was conducted with asymptomatic participants, participants with lower-limb injury and those with medial knee osteoarthritis. Spearman rank determined relationships between knee injury and outcome scores and hip and knee kinetic/kinematic gait parameters. K-Nearest Neighbour algorithm was used to determine which of the evaluated parameters created the strongest classifier model.

**Findings:**

Differences in outcome scores were evident between groups, with knee quality of life correlated to first and second peak external knee adduction moment (0.47, 0.55). Combining hip and knee kinetics with quality of life outcome produced the strongest classifier (1.00) with the least prediction error (0.02), enabling classification of injured subjects gait as characteristic of either asymptomatic or knee osteoarthritis subjects. When correlating outcome scores and biomechanical outcomes with osteoarthritis severity only maximum external hip and knee abduction moment (0.62, 0.62) in addition to first peak hip adduction moment (0.47) displayed significant correlations.

**Interpretation:**

The use of predictive models could enable clinicians to identify individuals at risk of knee osteoarthritis and be a cost-effective method for osteoarthritis screening.

## Highlights

• Individuals with lower limb injury have increased a risk of early on-set osteoarthritis.
• Current diagnosis techniques only identify established osteoarthritis.
• Biomechanical and self-reported questionnaires may be strongly correlated in gait.
• Prediction models could aid clinicians identify risk of osteoarthritis during gait.
• Self-reported questionnaires have low correlation to radiographic knee scans.

## 1 Introduction

Knee osteoarthritis (OA), a chronic degenerative joint disease, is a major cause of pain and disability creating a huge and continuously growing burden on individuals and society (Creaby et al., 2012, Kaufman et al., 2001, Mundermann et al., 2005). Knee OA is characterised by slow progression, with clinical diagnosis only possible at a late stage of the disease (Glyn-Jones et al., 2015). Therefore modifying interventions to slow and palliate disease advancement are limited if any, leaving joint replacement the mainstay of care. Early disease detection, however, could allow for a larger window of opportunity during which mitigating action could be taken before the onset of irreversible changes and aggravating disabilities (Chu et al., 2012).

Radiographic techniques are conventionally employed in the diagnosis of OA despite a poor correlation between radiographic findings and symptoms, and their ability to identify only the advanced stages of knee OA (Chu et al., 2012, Glyn-Jones et al., 2015). If the burden of OA is to be reduced, novel approaches for early clinical detection need to be identified. Magnetic Resonance Imaging (MRI) is sensitive in detecting structural changes in the knee joint, far exceeding that of conventional radiographs (Guermazi et al., 2009, Wirth et al., 2011) suggesting their use for early detection. However, with MRI techniques costing in the region of £400–£500 per scan it makes them unsuitable for large scale clinical trials and clinical translation. Recent research has explored using OA biomarkers, whilst these have shown promise their routine use remains a distant prospect (Glyn-Jones et al., 2015).

Less attention has been paid to the use of biomechanical markers of early OA. These can be assessed during gait analysis sessions and can typically be conducted at lower cost to MRI's (Patrick, 2003) and unlike MRI's, both legs can be analysed at once. Previous studies have shown characteristic patterns of knee OA, particularly at a late stage (Kaufman et al., 2001). However differences can also be appreciated in early OA with findings of asymmetrical weight distribution during sit-to-stand, as well as postural deficits and altered hip adduction moments during one-leg standing (Duffell et al., 2013, Duffell et al., 2014b). Astephen et al. (2008) highlighted how biomechanical mechanisms at the hip, knee and ankle were important when discriminating between individuals with moderate to severe knee OA. It is difficult however, from the cross-sectional nature of the study to infer if changes were due to disease progression or compensatory behaviours.

Individuals with a history of lower limb and knee injury have been found to have a four-fold increased risk of developing knee OA, with diagnosis occurring approximately 10 years earlier (Driban et al., 2014, Muthuri et al., 2011). Assessment of movement biomechanics in this group may prove useful in identifying early mechanical changes associated with knee OA development, allowing us to determine if early abnormalities that are characteristic of knee OA can be detected in injured “high risk” populations, thereby improving early diagnosis of OA allowing for early treatment strategies and ultimately OA prevention.

This study aims to identify the biomechanical parameters which are associated with functional and quality of life outcomes (knee injury and osteoarthritis outcome score (KOOS)) in OA and injured groups. In addition we will investigate whether KOOS outcomes and biomechanical parameters that are characteristic of knee OA can be used to predict early OA onset in injured populations. Finally we will explore whether a relationship exists between KOOS and biomechanical parameters in relation to radiographic knee OA severity.

## 2 Methods

### 2.1 Participants

This study was approved by the South West London Research Ethics Committee. All participants gave written informed consent prior to taking part.

The study included: 84 asymptomatic participants (control group), 41 with clinical and radiographic evidence of medial compartment knee OA (OA group; defined as a minimum 15–25% joint space narrowing in the medial compartment of their diseased knee (Duffell et al., 2014b)), and 51 participants with a history of musculoskeletal lower limb injury/surgery (injury group; fracture of the femur, knee or lower part of the leg, previous knee ligament, tendon, or meniscus injury). Due to the exploratory nature of the study, retrospective power calculations conducted post-testing indicated that a sample size of 12 for each of the control, injury and OA groups would give the study a power of 0.9. Both OA and injury participants were separated into unilateral (U-OA (31), U-I (41)) and bilateral (B-OA (10), B-I (10)). OA participants were grouped as unilateral or bilateral based on the number of knees presenting clinical symptoms and joint space narrowing as determined from radiographic images (confirmation by a consultant radiologist). Similarly, injured participants were grouped based on previous GP diagnoses of lower-limb injury. Control and injured participants were recruited from staff and students from Imperial NHS Trust and Imperial College London and posters circulated in hospitals/gyms/local health centres. OA participants were recruited from Imperial NHS Trust and local district regional hospitals.

Participants were excluded if they had neurological, rheumatoid or other systemic inflammatory arthritis, a body mass index (BMI) of > 35 kg/m^2^ or had undergone previous surgical treatment for knee OA. Participants were also excluded from the OA groups if they demonstrated other musculoskeletal conditions, were currently taking pain medication or were receiving treatments such as corticosteroid or hyaluronic injections. Knee joints for OA participants were scored for Kellgren and Lawrence (K-L) grade (0–4) from their most recent clinical radiographs (Kellgren and Lawrence, 1957).

### 2.2 Experimental protocol

A 10 camera Vicon motion capture system (T160, Vicon Motion System Ltd., Oxford, UK) and two portable force plates (Kistler Type 9286B, Kistler Instrumente AG, Winterthur, Switzerland) were used to collect joint kinematics and kinetics as participants walked along a 6 m walkway. Using the protocol described by Duffell et al. (2014a), twenty-three 14 mm diameter retro-reflective markers were positioned on participants' thorax, pelvis and lower limbs with four clusters of three markers positioned on participants' left and right thigh and calf segments; from these joint centres and anatomical frames were defined. Motion capture and force plate data were synchronized and captured at 100 Hz and 1000 Hz respectively. Participants walked 5 times barefoot at self-selected speed.

### 2.3 Self-reported outcomes

Participants completed a KOOS questionnaire (Roos et al., 1998) to assesses knee health in relation to 5 outcomes, with higher scores indicating less severe symptoms.

### 2.4 Data analysis

Motion capture and force plate data were processed using the methods described in Duffell et al., 2014a. Kinematic and kinetic parameters of the hip and knee joints in the sagittal and coronal planes and vertical ground reaction force (GRF) data were calculated (Table 1). Kinematic outputs were time normalised to 100% of the gait cycle, and kinetics to 100% of stance phase; heel strike and toe off were identified based on a 40 N vertical GRF threshold (Duffell et al., 2014b). Joint moments were normalised to body mass and height and expressed as external moments. Data analysis was performed using Matlab (TheMathWorksInc., Natick, MA, USA).

**Table 1:** Kinetic and kinematic discrete variables measured during gait.

Variable	Definition
Ground reaction force	
Maximum vertical force	Maximum vertical ground reaction force during the 1st 50% of the stance phase
Maximum vertical loading rate	Maximum slope of the vertical ground reaction force during the 1st 10% of the stance phase

Hip	
First peak rotation angle	Maximum vertical hip rotation angle during the stance phase of the gait cycle
Flexion angle RoM	Maximum hip angle calculated from maximum flexion to maximum extension during gait cycle
Abduction/adduction angle RoM	Maximum hip angle calculated from maximum hip abduction to maximum hip adduction during the gait cycle
Maximum external abduction moment	Maximum abduction moment of the hip during the 1st 20% of the stance phase
First peak external adduction moment	Maximum adduction moment of the hip during the 1st 50% of the stance phase
Second peak external adduction moment	Maximum adduction moment of the hip during the 2nd 50% of the stance phase

Knee	
First peak flexion angle	Maximum flexion angle during the stance phase of the gait cycle
Second peak flexion angle	Maximum flexion angle during the swing phase of the gait cycle
Flexion angle RoM	Maximum knee angle calculated from maximum flexion to maximum extension during the gait cycle
Maximum abduction moment	Maximum abduction moment of the knee during the 1st 20% of the stance phase
First peak external adduction moment	Maximum adduction moment of the knee during the 1st 50% of the stance phase
Second peak external adduction moment	Maximum adduction moment of the knee during the 2nd 50% of the stance phase

RoM = Range of Motion.

### 2.5 Statistical analysis

Statistical analysis was conducted using SigmaPlot (Version 11.0, Systat Software Inc.). All data was tested for normality and equality of variance using the Shapiro-Wilks and Levene's test. For age, height, mass, BMI, years since injury occurrence and OA diagnosis, biomechanical outcomes and gait velocity, One-Way ANOVA was used to assess statistical differences between all experimental groups. If variables were statistically significant a Bonferroni post-hoc test was performed. Mann-Whitney U analysis was used to assess statistical differences in self-reported outcomes. Significance was set at *P* < 0.05.

To reduce bias towards using biomechanical data of one leg over the other and to ensure biomechanical data of both legs of control, B-I and B-OA groups could be combined, *t*-tests were conducted on the kinematic and kinetic variables for these groups. Where data between legs was non-parametric a Mann-Whitney U analysis was used. B-I and B-OA cases were then combined with data from U-I and U-OA to create an injured and OA group for use within the prediction model analysis. Spearman rank correlation coefficient calculated the relationship between KOOS and biomechanical parameters (Bekkers et al., 2009).

For a subset of 18 OA subjects, representing participants with radiographs within 1 year prior to testing, continuous biomechanical and KOOS parameters were transformed into ranked data (Nahm, 2016), enabling Spearman's rank correlation coefficient to be used to calculate their relationship with K-L grade (Hauke and Kossowski, 2011).

To identify the importance of biomechanical parameters and KOOS outcomes during gait, feature selection was carried out on all variables using a minimum-redundancy maximum-relevancy method (Khushaba et al., 2011, Peng et al., 2005). This method aims to select features that best represent the target classification variable whilst being as different as possible from each other (minimum redundancy) but highly correlated to the classification variable (maximum relevancy). For more details please refer to Peng et al., 2005.

### 2.6 K-Nearest Neighbor classifier

K-Nearest Neighbor (K-NN) was used to classify whether self-reported outcomes and biomechanical parameters of injured participants were characteristic of control or OA. Reference to injured and OA groups hence forth with regards to the K-NN approach uses the combined data of U-I with B-I and U-OA with B-OA.

K-NN classifies a case, in this instance an injured individual, as either control or OA based on features included in the K-NN model, using a majority vote of its K-Nearest Neighbor (Zhu et al., 2007). The number of models created were defined by the rank of the final KOOS outcome during feature selection. A total of 12 training models were created, each including one of the ranked parameters. To be able to combine KOOS outcomes and biomechanical parameters into additional K-NN models, all biomechanical parameters were normalised.

$$X{'}_{i}=\frac{\left(X_{i}-X_{\min}\right)\times \left(L_{\max}–L_{\min}\right)}{\left(X_{max}-X_{min}\right)}+L_{\min}$$

where *X* is the raw data, *X*_min_ and *X*_max_ are the smallest and highest value, with *L*_min_ and *L*_max_ the lower and upper limit of the new output range. *X*′_*i*_ is the transformed data (Begg and Palaniswami, 2006).

Eighty-eight participants' KOOS and biomechanical data, from control and OA groups were randomly selected for training, with the remaining 37 participants selected for testing the models (70% training/30% test). Training the models with OA data was deemed appropriate to classify injured individuals at higher risk of developing secondary OA, as individuals with primary OA irrespective of previous knee injury are predisposed to secondary OA, both of which exhibit the same pathology (Doherty et al., 1983).

The Euclidean distance metric was chosen to quantify similarity between data points. The number of neighbors (K) which output the smallest re-substitution loss fraction (Rloss) and prediction error (Kloss) was assessed using cross-validation (Zhu et al., 2007). The area under the Receiver Operating Characteristic (ROC) curve was then recorded to measure each model's performance (Fawcett, 2004). Model performance was assessed using the average error of Rloss and Kloss, any models with the same average error rates, the area under the curve (AUC) and false classification percentage were assessed.

## 3 Results

One-Way ANOVA indicated a significant difference for age, weight BMI and gait velocity between groups (Table 2). Bonferroni post hoc analysis (*P* ≤ 0.05) indicated that U-OA and B-OA were significantly older than control, U-I and B-I. U-OA and B-OA also had a significantly greater body mass than controls and a significantly greater BMI in comparison to the controls, U-I and B-I. Gait velocity was significantly reduced for U-I, U-OA and B-OA compared to control.

**Table 2:** Mean (SD) demographics for control, injured and OA groups.

Variable	Control (n = 84)	U-I (n = 41)	B-I (n = 10)	U-OA (n = 31)	B-OA (n = 10)
Sex (male/female)	39/45	26/15	6/4	17/14	5/5
Age (years)	44.8 (16.5)	44.5 (15.8)	46.3 (15.5)	56.8 (10.5)	59.8 (13.8)
Height (cm)	170.74 (10.28)	173.85 (9.29)	174.18 (8.66)	169.66 (11.43)	170.82 (13.13)
Mass (kg)	68.32 (12.38)	71.85(11.4)	73.93 (12.17)	75.73 (12.00)	80.46 (23.60)
BMI (kg/m^2^)	23.3 (3.1)	23.7 (2.4)	24.1 (3.2)	26.3 (3.4)	27.0 (4.4)
Injury occurrence (years prior to testing)^a^	–	9 (13)	22 (17)	–	–
OA diagnosis (years prior to testing)^a^	–	–	–	3 (5)	2 (2)
K-L grade	–	–	–	3 (1)	3 (1)
Gait velocity (m/s)	1.25 (0.19)	1.19 (0.19)^b^	1.23 (0.19)	1.13 (0.21)^b^	1.11 (0.17)^b^

BMI = Body mass index, K/L grade = Kellgren-Lawrence grading scale (0–4).

a Mean injury occurrence and OA diagnosis (U-I, U-OA) calculated from randomised subject selection (N = 10, N = 10) to match B-I and B-OA subject data.

b Indicates significant difference in gait velocity compared to control.

Mann-Whitney U analysis of KOOS outcomes indicated that for pain, symptom, QOL and S/R; U-I, B-I, U-OA and B-OA had significantly worse scores than controls (Table 3). U-OA and B-OA also displayed significantly lower scores for each individual KOOS outcome than U-I and B-I. There were significantly lower scores for U-OA and B-OA in KOOS outcome ADL in comparison to ADL scores for control, U-I and B-I.

**Table 3:** Mean (SD) KOOS outcome score for control, injured and OA groups.

KOOS outcome (0 − 100)	Control	U-I	B-I	U-OA	B-OA
Pain	95.3 (8.0)	85.0 (14.7)	83.1 (17.0)	61.9 (19.2)	56.5 (17.0)
Symptoms	91.7 (8.0)	77.2 (18.5)	78.2 (19.7)	54.0 (21.6)	60.3 (22.5)
ADL	97.5 (6.1)	92.8 (11.7)	90.3 (15.2)	68.0 (19.9)	67.7 (21.5)
QOL	92.1 (11.8)	63.18 (24.9)	59.4 (26.3)	38.1 (23.6)	28.9 (21.1)
S/R	94.6 (9.8)	78.15 (22.5)	72.0 (27.5)	46.0 (27.2)	36.1 (33.5)

KOOS = Knee injury and osteoarthritis outcome score, ADL = function in daily life, QOL = knee related quality of life, S/R = sport and recreation.

Analysis indicated that KOOS outcomes did not meet requirements of homogeneity, as such spearman rank assessed correlations. When analysing data for control, injured and OA groups together, there were significant correlations for ADL and QOL between all biomechanical parameters, apart from the first and second peak external hip adduction moments, as shown in Table 4. Biomechanical parameters most strongly correlated to ADL and QOL, were the first and second peak external knee adduction moments (KAM). This was a significantly (*P* ≤ 0.01) modest positive correlation, indicating that first and second peak external KAMs were greater when ADL and QOL outcome scores were higher.

**Table 4:** 1) Correlation coefficients of control, injured and OA groups between self-reported outcomes and biomechanical parameters; 2) correlation coefficients of K-L grade with self-reported outcomes and biomechanical parameters using a sub-set of the OA group.

*F* rank	Variable	Spearman rank correlation					
		1)					2)
		Pain	Symptom	ADL	QOL	S/R	K-L grade
1	Maximum external hip abduction moment (Nm/weight ∗ height)	− 0.23 ⁎⁎	− 0.24 ⁎⁎	− 0.27 ⁎⁎	− 0.22 ⁎⁎	− 0.27 ⁎⁎	0.62⁎⁎
2	S/R	0.89 ⁎⁎	0.81 ⁎⁎	0.87 ⁎⁎	0.87 ⁎⁎	–	0.21
3	First peak external knee adduction moment (Nm/weight ∗ height)	0.37 ⁎⁎	0.37 ⁎⁎	0.36 ⁎⁎	0.47 ⁎⁎	0.40 ⁎⁎	− 0.10
4	Second peak external knee adduction moment (Nm/weight ∗ height)	0.43 ⁎⁎	0.43 ⁎⁎	0.44 ⁎⁎	0.55 ⁎⁎	0.46 ⁎⁎	0.29
5	Symptom	0.81 ⁎⁎	–	0.80 ⁎⁎	0.79 ⁎⁎	0.81 ⁎⁎	− 0.27
6	Maximum vertical loading rate (Nm/s/weight ∗ height)	0.22 ⁎⁎	0.17 ⁎	0.24 ⁎⁎	0.18 ⁎	0.22 ⁎⁎	0.14
7	First peak external hip adduction moment (Nm/weight ∗ height)	0.02	0.01	− 0.04	0.10	0.02	0.47⁎
8	Maximum external knee abduction moment (Nm/weight ∗ height)	− 0.23 ⁎⁎	− 0.24 ⁎⁎	− 0.22 ⁎⁎	− 0.28 ⁎⁎	− 0.26 ⁎⁎	0.62⁎⁎
9	QOL	0.89 ⁎⁎	0.79 ⁎⁎	0.80 ⁎⁎	–	0.87 ⁎⁎	0.21
10	Second peak external hip adduction moment (Nm/weight ∗ height)	− 0.01	− 0.02	− 0.01	0.7	− 0.01	0.38
11	ADL	0.89 ⁎⁎	0.80 ⁎⁎	–	0.80 ⁎⁎	0.87 ⁎⁎	0.00
12	Pain	–	0.81 ⁎⁎	0.89 ⁎⁎	0.85 ⁎⁎	0.89 ⁎⁎	0.01

*F* rank = variable importance based on minimum-redundancy maximum-relevancy feature selection; Spearman rank = ≤ 0.39 (weak), 0.40–0.59 (modest), 0.60–0.79 (strong), ≥ 0.8 (very strong).

⁎ P = < 0.05.

⁎⁎ P = < 0.001.

Mann Whitney U analysis indicated no statistical differences for biomechanical parameters between U-I and B-I nor between U-OA and B-OA, as such data was combined creating an injured and OA group for K-NN models. Table 5 contains error rates and performance strengths of each K-NN model for the top 12 variables identified from feature selection. Based on the average Rloss and Kloss, comparisons of the KOOS outcomes indicated that QOL had the lowest error rate of 0.07 (0.00) with an AUC of 0.98 out of a possible 1.00, signifying the model had high classifier performance. This is shown by only 4% of control participants falsely classified with no false OA classifications. When comparing the biomechanical models, the second peak external KAM had the lowest error rate of 0.08 (0.02) with the highest AUC of 0.95, resulting in 4% of control subjects and 9% of OA participants falsely classified.

**Table 5:** K-Nearest Neighbor model re-substitution loss, prediction loss and performance strength during testing.

*F* rank	Training model	Rloss	Kloss	Average loss	ROC (AUC)	False classification (%)	
						Control	OA
1	Maximum external hip abduction moment	0.17	0.41	0.29 (0.17)	0.47	26	91
2	S/R	0.10	0.10	0.10 (0.00)	0.82	0	36
3	First peak external knee adduction moment	0.06	0.10	0.08 (0.03)	0.88	8	18
4	Second peak external knee adduction moment	0.07	0.09	0.08 (0.02)	0.94	4	9
5	Symptom	0.10	0.10	0.10 (0.00)	0.95	0	9
6	Maximum vertical loading rate	0.24	0.25	0.24 (0.01)	0.74	15	36
7	First peak external hip adduction moment	0.23	0.36	0.29 (0.09)	0.41	62	46
8	Maximum external knee abduction moment	0.11	0.14	0.13 (0.02)	0.87	23	0
9	QOL	0.07	0.07	0.07 (0.00)	0.98	4	0
10	Second peak external hip adduction moment	0.24	0.38	0.31 (0.10)	0.62	4	73
11	ADL	0.11	0.11	0.11 (0.00)	0.92	12	9
12	Pain	0.08	0.13	0.10 (0.03)	0.79	15	27

	All self-reported outcomes	0.08	0.08	0.08 (0.00)	0.82	0	23
	All biomechanical parameters	0.02	0.02	0.02 (0.00)	0.92	8	9
	All biomechanical parameters and QOL outcome	0.01	0.03	0.02 (0.01)	1.00	0	0

When combining all KOOS outcomes the K-NN model displayed an error rate of 0.08 (0.00) with an AUC of 0.82, with all control correctly classified, yet 23% of OA participants falsely classified. The model including all biomechanical parameters had a lower error rate of 0.02 (0.00), an AUC of 0.92 resulting in 8% of control subjects and 9% of OA subjects being falsely classified. The K-NN model which displayed an equally low error rate of 0.02 (0.01) and had the largest AUC of 1.00, emerged from combining all biomechanical parameters with QOL. This resulted in no false classification of control or OA participants.

**Fig. 1:**
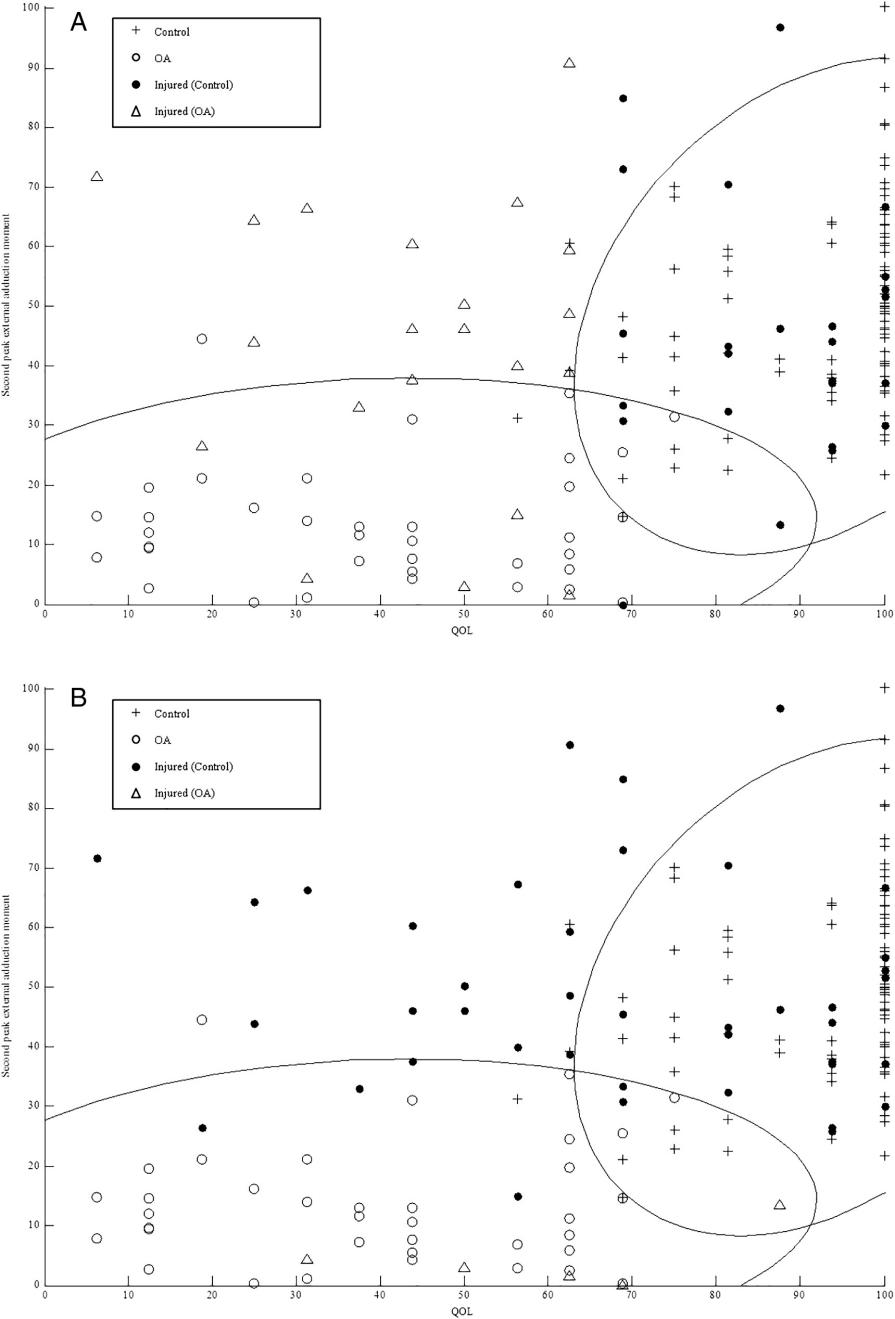

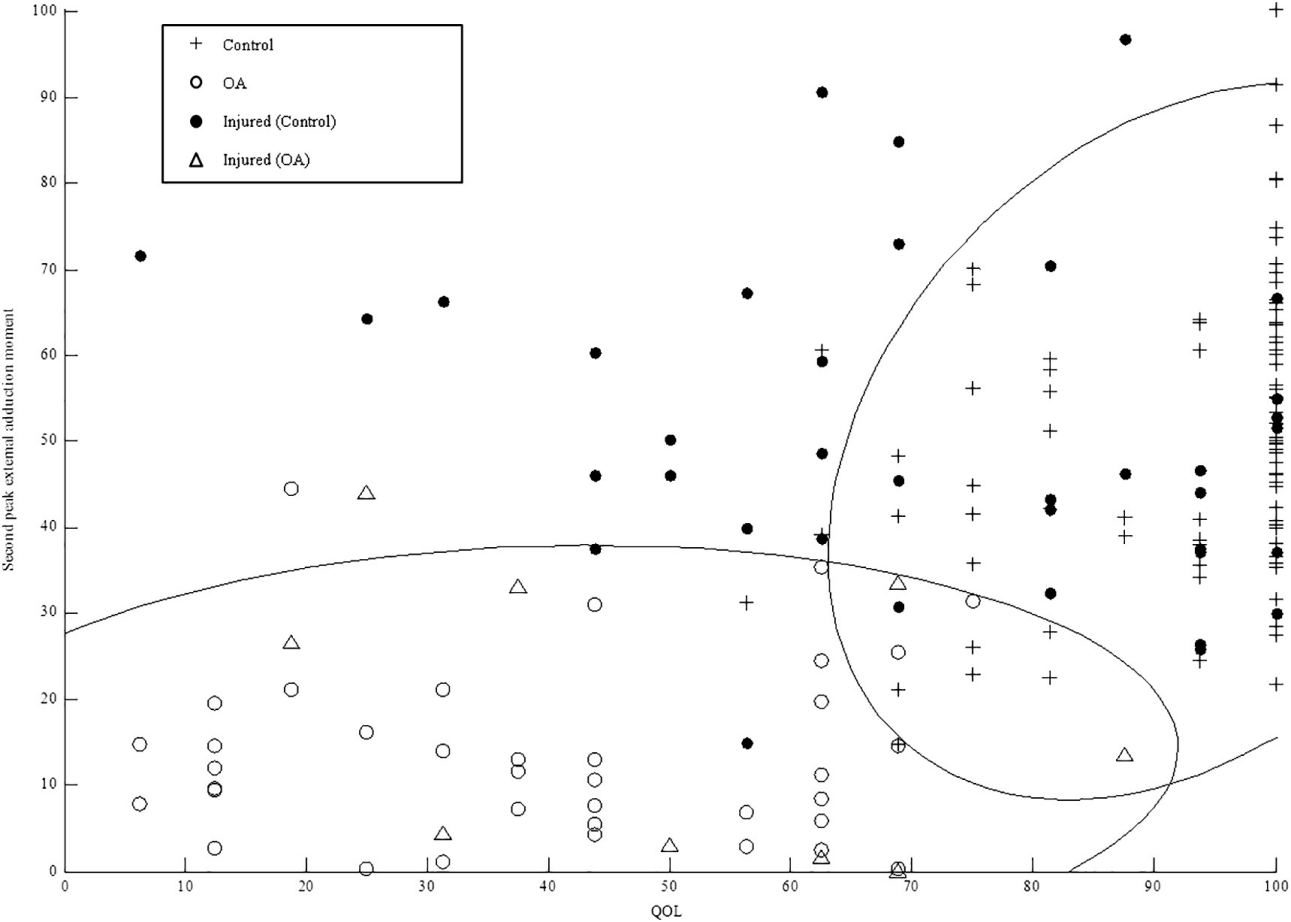
Quality of life K-Nearest Neighbour model predicting injured individuals gait as characteristic of control or OA (a); all biomechanical parameter K-Nearest Neighbour model predicting injured individuals gait as characteristic of control or OA (b); all biomechanical parameters plus quality of life K-Nearest Neighbour model predicting injured individuals gait as characteristic of control or OA (c).

Fig. 1a–c shows control and OA group's QOL in relation to their second peak KAM, as these were the strongest correlated KOOS and biomechanical parameters as indicated by the Spearman rank. As individual's QOL score increased their second peak KAM also increased. There were also no control participants with a QOL below 60 and no OA participants with a QOL higher than 80. Based on the parameters used as an inputs, prediction of injured participant's gait as either control or OA varied between models.

When running the QOL model (Fig. 1a), 21 of the 51 injured participants were predicted as OA, 6 of which were within the OA groups 95% Euclidean error interval. The model, which used all biomechanical parameters (Fig. 1b), predicted 5 injured participants as OA, all of whom were within the OA group's 95% Euclidean error interval. The strongest performing model, which combined all biomechanical parameters with QOL (Fig. 1c), predicted 9 injured participants as having biomechanical and QOL characteristics of OA, 8 of which were within the OA group's 95% Euclidean error interval. Whilst differences are evident between model predictions, across all models the same 3 participants were predicted as OA. In addition across both the QOL and the biomechanical model, a further three participants were predicted as OA; as well as another two injured participants predicted across the biomechanical and final model.

Finally, Spearman rank of KOOS outcomes with K-L grade for the subset of 18 OA participants are shown in Table 4. This indicated that pain, ADL, QOL and S/R were positively correlated, with symptom negatively correlated to K-L grade. No significance was found. When correlating K-L grade to biomechanical parameters, the maximum external hip abduction moment and maximum external knee abduction moment were found to have a significant (*P* ≤ 0.01) strong positive correlation with K-L grade. Similarly the first peak hip adduction moment had a significant (*P* = 0.05) modest positive correlation with K-L grade.

## 4 Discussion

This study investigated whether KOOS outcomes are associated with biomechanical gait parameters. Moreover, an algorithm is proposed based on KOOS outcomes and biomechanical gait parameters to predict gait abnormalities characteristic of knee OA in injured populations, with the view of using it for early detection of knee OA. Finally, relationships between KOOS and biomechanical gait parameters in relation to OA severity were explored.

The findings showed positive correlations between KOOS outcome scores for control, injured and OA groups, specifically ADL and QOL with biomechanical parameters of the knee, particularly the first and second peak external KAMs (Table 4). The external KAM has been associated with the presence and progression of knee OA severity (Kang et al., 2014), with evidence suggesting it reflects increased medial tibiofemoral compartment compressive load (Schipplein and Andriacchi, 1991, Winby et al., 2009). Increased pain due to compressive loading associated with OA, can influence how individuals function and perform everyday tasks (Hartwick et al., 2003, Heidari, 2011), demonstrated in the relationship found between increasing pain and decreasing ADL and QOL. Our results also indicate that as painful symptoms increase, the first and second peak external KAMs decrease. This is possibly evidence of compensatory reductions of gait velocity in OA individuals, in an attempt to reduce painful symptoms (Mundermann et al., 2004). This agrees with Zeni and Higginson (2009) who found knee moments to be significantly lower when individuals walked at freely chosen velocities.

In this study both OA and injured groups displayed significantly reduced scores for pain, symptom, QOL and S/R in comparison to controls. This is in line with previous studies who found that 12 years after ACL rupture 75% of female soccer players reported significant symptoms affecting knee QOL, with 42% having symptomatic radiographic knee OA (Lohmander et al., 2004). Similarly 15–20 years after meniscectomy 50% of patients had reported OA, with QOL post-surgery significantly worse than age-matched controls (Englund et al., 2001, Englund et al., 2003). Reductions in meniscal surface area can potentially increase contact pressures in regions unaccustomed to weight bearing loads by up to 65% (Brindle et al., 2001) resulting in compensatory movements and decreases in knee movement (Cattano et al., 2013).

The KOOS and biomechanical parameters reported in our study highlight how factors such as knee health, pain, age and body mass which are linked to OA progression can affect gait; alterations in gait biomechanics can then in turn decrease knee health and facilitate OA progression (Chang et al., 2007, Cooper et al., 2000). This intricate link between factors makes the prediction of knee OA using traditional diagnosis approaches problematic. As a result, we also investigated whether some of the factors associated with OA could be used with machine learning algorithms to detect subtle changes in gait to predict knee OA in at risk populations. The use of K-NN algorithm has been shown to be a better predictor of rehabilitation potential in home-care clients than current clinical assessment protocols, enhancing clinical decision making (Zhu et al., 2007).

Comparisons of our K-NN models illustrates that QOL, which this study has shown to be correlated to peak KAMs and OA severity, produces a K-NN model with greater classifier accuracy and strength compared to one which uses all KOOS outcomes (Table 5). The clinical application of using our QOL K-NN model is it is a feasible, inexpensive screening method in comparison to MRI scans enabling clinicians to periodically track and identify worsening knee QOL in at risk individuals.

When using the model containing all biomechanical parameters combined with QOL to classify control and OA subjects, no false classifications were obtained. When inputting injured individuals into the model, each individuals gait was classified as characteristic of healthy or OA. Although this model showed better performance than the QOL model, it may present with the difficulty of having biomechanics measures taken within clinical environments. For these reasons the translation from biomechanical measures to more easily quantifiable parameters will be explored. This way clinical use can be ensured without limiting the classification to the KOOS QOL, which still remains a subjective measure.

One way could be to investigate whether QOL in combination with functional knee joint performance tests, as well as parameters of easy access in clinical environment such as, step length or walking speed, could be used in a K-NN model to support clinicians during assessments in clinics. Holsgaard-Larsen et al. (2014) found that in patients with ACL reconstructions, there were moderate associations between one-legged distance hopping, knee extensor and flexor maximum voluntary contraction and QOL when using a regression analysis. The availability of a predictive model incorporating such parameters could provide clinicians with unique information for managing patients (Willke et al., 2004), enabling accurately identification and tracking of individuals who would benefit from exercise interventions or those requiring further biomechanical gait assessments. In addition, the K-NN model could be presented in graphical form, enabling patients to better monitor and understand their knee health. Overall results of this study show how machine learning techniques could have clinical applications by helping diagnosis and assessment of those with knee problems.

Whilst the models developed showed excellent prediction accuracy during testing and classified new injured patients, future work should focus on validation of models using follow-up MRIs of injured individuals whom the models predict as displaying OA gait characteristics. Successful validation could lead to an alternative method over that of MRI's for screening individuals before onset or further OA progression. Research has previously shown treatment efficacy and societal cost-effectiveness of gait assessments in reducing incidence of surgery (Wren et al., 2009, Wren et al., 2011). If such models were combined with commercially available and comparatively inexpensive gait assessments, they may allow individuals to more frequently monitor their knee health, allowing for interventions aimed at reducing the total cases and cost of knee OA in at risk populations.

When correlating self-reported outcomes with OA severity, there were no significant correlations between KOOS outcomes and OA severity, which is in line with previous research (Kahn et al., 2013, Muraki et al., 2009). When correlating biomechanical parameters with OA severity, the maximum external hip abduction moment and maximum external knee abduction moment had the strongest positive significant correlation with K-L grade. The suggestion of an increasing maximum hip abduction moment as OA severity increases, supports previous research which found that individuals with mild to moderate knee OA display greater hip abduction moments during gait compared to control (Briem and Snyder-Mackler, 2009, Mundermann et al., 2005). Our findings support earlier work that indicated that increased hip moments through lateral trunk sway towards the healthy limb, aids in reducing the mediolateral distance between centre of mass and the knee joint during early stance, reducing knee and hip medial forces (Andriacchi and Mundermann, 2006, Briem and Snyder-Mackler, 2009).

In contrast to Zeni and Higginson (2009) who found no differences in joint moments between control and OA patients when walking at a controlled speed, our study groups were able to walk at their freely chosen speed, in an effort to mirror everyday gait. As such injured and OA groups walked slower in comparison to controls. The differences therefore seen in the first and second peak external KAM's and strength of correlations could be attributed to the differences in walking speeds between groups (Silvernail et al., 2013). Whilst potentially a source of bias within this study, the self-selected walking design was chosen to enable our predictive models to be trained with biomechanical data representative of natural gait and current clinical practice. It is possible that if our models were trained with data from controlled gait velocity, the ability of the models to effectively classify at risk individuals during clinical gait assessments may have been adversely affected (Vail and Veloso, 2004).

It should also be acknowledged that control and injured participants in this study did not have radiographs; therefore it is possible that these cohorts could have had morphological signs of knee OA without clinical symptoms (Beattie et al., 2005). In addition, only 18 OA subjects had radiographs taken a year prior to testing, of which only the medial compartments were used to classify OA severity. Whilst Sward et al. (2009) found that structural changes in non-traumatic OA patients predominantly occur in the medial compartment, future research should focus on the lateral side, as post-traumatic patients were found to have structural changes evenly distributed between medial and lateral sides. Groups were also not matched for body mass or age, with U-OA and B-OA having a greater body mass and older than control and injured.

Such differences in OA group's body mass and age may have influenced the observed biomechanics and walking velocities between groups. Indeed Silvernail et al. (2013) showed that whilst overweight young individuals had similar knee biomechanics, gait velocities were slower compared to normal weight young adults. This is further illustrated by Messier (1994) who found that being older affects both gait biomechanics and velocities in individuals with knee OA. Finally, average time since injury was not controlled. Future consideration should be given to undertaking MRI scans of healthy and injured individuals at the time of testing.

## 5 Conclusion

This exploratory study has found that individuals with lower-limb injury and knee OA have lower KOOS scores than asymptomatic individuals. The relationship present between peak KAMs, ADL and QOL also supports previous evidence suggesting that worsening knee QOL and knee functionality are linked to peak KAMs, particularly in individuals who have undergone knee surgery (Ilich et al., 2009). This gives further credence to the idea that KOOS scores related to peak KAMs during gait are a sensitive measure for predicting those at risk of developing poor knee function over time and could be used in a clinical setting. Additionally our findings that KOOS outcomes are not significantly correlated to OA severity yet are positively related to biomechanical parameters, strengthens the idea that alternative diagnosis methods may be effective compared to just using self-reported or clinical symptoms and provide another option over costly MRI's.

To this end we have demonstrated how algorithms such as K-NN models could be a viable cost-effective method if used in conjunction with biomechanical gait analysis sessions for screening injured individuals at risk of developing early knee OA. Such models could provide a cheaper alternative to monitor knee health in comparison to MRI's, which can be of great expense to patients and health services. Whilst the only way to prevent OA after injury is to prevent the injury from occurring in the first place and whilst future work is required to investigate the effectiveness of the K-NN models highlighted in this study with age and mass matched individuals, early indications are that models such as the one presented in this paper could be used to assist clinicians to predict individuals at high risk of early onset knee OA from injured populations. This could then potentially help clinicians to reduce the effects OA has on individual's quality of life and speed of disease progression.

## Conflict of interest

The authors declare no conflicts of interest.
